# Changes in the Scientific Information Environment During the COVID-19 Pandemic: The Importance of Scientific Situational Awareness in Responding to the Infodemic

**DOI:** 10.1089/hs.2020.0194

**Published:** 2021-02-18

**Authors:** John K. Iskander, Katherine M. Bianchi

**Affiliations:** John K. Iskander, MD, is Senior Advisor and Katherine M. Bianchi is a Fellow; both in the Office of the Surgeon General (OSG), Office of the Assistant Secretary for Health, Department of Health and Human Services (HHS), Washington DC. John K. Iskander is also a Captain, United States Public Health Service. This work does not represent the official position of the OSG or HHS. Names of institutions, companies, and products are provided for identification purposes only and do not imply any endorsement by the OSG or HHS.

**Keywords:** COVID-19, Epidemic management/response, Citizen engagement, Public health preparedness/response, Situational awareness

## From Situational Awareness to Scientific Situational Awareness

Public health response to the coronavirus disease 2019 (COVID-19) pandemic has highlighted the need for situational awareness.^1^ In particular, scientific situational awareness is required to formulate science-informed policies. In public health emergency response, situational awareness typically comprises public health surveillance and laboratory testing data, but also includes information on relevant health or public health infrastructure, population data, and environmental exposure data that may be analyzed and displayed spatiotemporally.^2^ A general definition of scientific situational awareness, or awareness of the scientific information environment, may include monitoring citation databases and newer repositories such as preprint servers for publication of new findings and sharing relevant references within organizations or emergency response structures.^3^

In this commentary, we briefly describe ways in which the scientific information landscape has evolved in an accelerated manner during the COVID-19 pandemic and propose ways for individuals and organizations to maintain and contribute to enhanced scientific situational awareness as a means of responding to the COVID-19 infodemic. The current pandemic has seen the creation or accelerated use of new platforms or repositories for scientific data and information and new scientific publishing models and article forms, resulting in a mixture of new and old challenges to the integrity of available scientific information exchange forums. Examples of each category of novel or expanded scientific information resources follow.

The 2009 H1N1 influenza pandemic occurred after the rise of the internet. The decade since then has seen major trends in the digital world, such as the application of artificial intelligence and “big data” methodologies including machine learning to public health.^4^ The COVID-19 pandemic has been the first to fully leverage big data tools, for example, in the form of interactive dashboards that aggregate data and track various global metrics related to the pandemic. In addition to the widely used global case data dashboard created by a multidisciplinary team at Johns Hopkins University,^5^ newer dashboards track severe acute respiratory syndrome coronavirus 2 (SARS-CoV-2) seroprevalence studies^6^ and COVID-19 related clinical trials.^7^

During the COVID-19 pandemic, newer types of repositories of scientific information have achieved increased prominence among the public health and research community and have also reached public prominence through media coverage. We summarize key features of some of these platforms in Table 1. These include preprint servers such as bioRχiv and medRχiv, which share scientific reports prior to formal peer review, and websites such as GitHub, which hosts computer programming code for specific research projects. Additional notable features of GitHub are its use as a platform for collaboration and teaching and its encrypted, secure nature.^8^ While the limitations of nonpeer-reviewed scientific information may seem obvious, it is worth remembering that early in the pandemic several prominent peer-reviewed papers were retracted by major journals when source data could not be independently validated.^9^ Clinical trial registration databases, including clinicaltrials.gov, are another type of scientific information resource that are not new but are receiving expanded attention and being discovered by broader audiences seeking information about trials involving COVID-19 vaccines and therapeutics. Newer users may not be aware of the well-documented limitations of trial databases, including incompleteness of information provided.^10^

**Table 1.** Selected Types of Scientific Repositories and Platforms

Type	Examples	Description
Traditional citation databases	PubMed, PubMed Central, Scopus, Web of Science	Peer-reviewed literature, including original research, systematic reviews, meta-analyses, and review articles
Preprint servers	Rχiv, BioRχiv, MedRχiv	Preprints share scientific data and analysis prior to formal peer review
Data dashboards	Johns Hopkins University, *New York Times*, USAFacts, WorldoMeter, COVID Tracking Project	Aggregated national and/or international data on COVID-19 case counts, morbidity, and mortality
Source code and data repositories	GitHub, PsychArchives	Host programming code for research projects; some may include datasets
Clinical trial databases	Clinicaltrials.gov	List details for clinical studies; information may be incomplete

The scientific publishing landscape has continued its evolution during the time of the pandemic, and models combining traditional editorial and newer publishing practices have become more prominent. In addition to preprint servers, traditional journals may similarly post articles prior to peer review or may facilitate the posting of papers as preprints while they are simultaneously undergoing peer review. While it has frequently been the case that major journal publishers offer open access to related content early on during public health emergencies, what has been notable during the time of COVID-19 has been the sustained commitment of multiple major academic publishers to enhanced open access. Newer article formats such as “living,” rapid, and narrative reviews^11^ have taken an increasing role among COVID-19 scientific publications compared to traditional systematic reviews, due to their greater speed of completion and dissemination and their ability to serially incorporate rapidly emerging information.

Unsurprisingly, scientific publishing and information sharing has not been immune to the misinformation and disinformation aspects of the infodemic. Certain challenges to credibility may be accelerating as a consequence of COVID-19. Some accountability researchers have asserted that the rate of retractions has been higher during the current pandemic, while others have disputed those findings.^12,13^ Nevertheless, the need for vigilance in addressing information found to be inaccurate or fraudulent, even after peer review and publication in prestigious journals, will remain a core part of maintaining scientific situational awareness. The monitoring of websites such as Retraction Watch can be an important part of information surveillance. New forms of scientific content may bring new risks as well. Preprints may be appropriated by social media users as a platform for spreading ideological messages that are separate from the scientific content of the paper.^14^

## Recommendations to Promote Scientific Situational Awareness

Building capacity for scientific situational awareness begins with the realization that the scientific and public health communities are both producers and consumers of scientific information. There is a need to build literacy among consumers of the new scientific information environment, recognizing that those users now go beyond the traditional research and policymaking community to include journalists and the public (including citizen scientists). In the following section, we first describe some general principles that underlie good scientific situational awareness practices and then provide examples of specific strategies that can be used to build robust scientific situational awareness capacity.

### Favor Consistency over Completeness

In developing individual or institutional approaches to scientific situational awareness, consistency in retrieval of information, such as using search criteria that can be replicated, is probably more important than completeness. Public health surveillance systems, though subject to incomplete reporting, have proven useful to monitor trends in occurrence of and risk factors for a variety of diseases.^15^ Science Clips, a weekly current literature awareness serviced produced by the Stephen B. Thacker CDC Library and focused on CDC-authored publications, includes searches from 8 citation databases, using standardized search terms and article categories. It can be considered as a type of surveillance of the CDC publication environment and has proven useful for monitoring trends in numbers and types of publications.^16^ It is also worth remembering that searches, whether of the internet, citation databases, or other information sources can never be complete: rather they are categorized by library and information scientists as more or less comprehensive, based on the search criteria and the number and type of sources searched.

### Value Primary Sources or Original Data

Whenever possible, groups seeking scientific situational awareness should prioritize primary over secondary sources. In the modern information environment, this may mean not just seeking the original publications, rather than just the accompanying or referencing media reports, but also, in some cases, locating and analyzing the original datasets. This principle should apply to the retrieval and sharing of scientific information. Even if not required to, institutions and individual scientists can promote use of primary sources by others by publishing in open access formats, publishing both positive and negative findings, and making data and code publicly available, according to published best practices.^17^ Broader availability of primary data and source code may bring potentially flawed analyses to light sooner but can also promote collaboration and expansion of research capacity.

### Prioritize Accuracy not Primacy

In seeking scientific situational awareness or producing information to contribute to it, primacy, that is, being the first to report something, must be balanced against accuracy. This is true even during public health emergencies. It is not just that information published quickly may be wrong, but that such reports may provide an incomplete scientific picture and leave important questions unanswered. Consider the case of SARS-CoV-2 virus and reinfection. The first report of confirmed reinfection out of Hong Kong was accompanied by extensive scientific and media attention.^18^ However, many key issues about reinfection remain, including which epidemiologic or laboratory criteria should be used to define it and how frequently or infrequently it occurs.^19^ Ultimately, sharing scientific information is not just a matter of being first, but of providing knowledge that is accurate, relevant, and actionable. In recognition of this, some public health-focused journals do not permit authors to make primacy claims. Authors should not automatically seek to publish every “first” finding, especially if such findings are very narrow in scope and not likely to be immediately useful or important.

In evaluating “firsts” or other novel claims in published or preprinted literature, the so-called “Sagan standard”^20^ can be an important guiding principle. This is often stated as “extraordinary claims require extraordinary evidence.” In the context of this infodemic, we might add the element of transparency to this aphorism. Practically speaking, this means that data from previously unknown or undescribed sources or data that are unavailable for analysis or cannot be used to reproduce claimed findings should be suspect until verified or replicated. Findings from studies that lack control or comparison groups, while of some utility early on in describing clinical features of a new infection, should not be overgeneralized.

### Search Beyond Original Research

While developing strategies to review and synthesize the literature, scientists, communicators, editors, and other scientific curators should take into account that the peer-reviewed literature contains not only original data analysis, but also a broad variety of content, from sources such as public health surveillance reports, commentaries, and even policy proposals.^21^ Not all scientifically relevant information is published in preprints or peer-reviewed literature. Examples of “grey” literature include reports from the National Academy of Medicine or congressional legislation, such as the 21st Century CURES Act,^22^ as well as conference abstracts, posters, and other unpublished data. Awareness of unpublished scientific information may be especially important during this pandemic, as authors may face system-level barriers to publication.^23^

### Consider Curated Sources in Context

Curated information sources—any publication or content that has an editor or editorial content—can be very valuable, but users should be aware of who is doing the curating, whether any competing or conflicting interests are disclosed, and whether the curation involves selecting, featuring, or interpreting published or posted scientific information. Because of the huge quantities of peer-reviewed and preprint articles available, machine learning algorithms can be a valuable component of scientific information curation. However, systems that include a “human in the loop,” such as an editor, to review algorithmic outputs may useful for guarding against systematic biases.^24^

### Organize Scientific Situational Awareness in a Purpose-Built Way

Scientific situational awareness information retrieval, organization, and dissemination should be designed with the needs of internal or external users in mind. While journal editors need to monitor what content and which authors are being retracted from other journals, communications staff may be focused not just on communications from their organization, but also what is being said in social media or other channels about the organization. Additional systematic sources beyond traditional scientific citation databases (eg, LexisNexis or internal news clipping services) may need to be incorporated to monitor media or legal information environments associated with a particular public health emergency.

### Work Across Disciplines to Build Capacity

Just as in other aspects of the scientific response to COVID-19, collaboration on scientific situational awareness can have important benefits. The increasing diversity of scientific resources, from source code to dashboards to social media, suggests the importance of interdisciplinary collaboration in developing scientific situational awareness capacity.^5^ Scientific situational awareness teams may include computer scientists, informaticians, economists, medical or reference librarians, data scientists, and health communicators, in addition to clinical practitioners, epidemiologists, and laboratory scientists. The experience and expertise of reference librarians can be especially beneficial in conducting rapid or living reviews and retrieving information from the “grey” literature and less well-known citation databases. Having an interdisciplinary mixture of scientific skillsets can yield richer raw information and more useful syntheses. Table 2 summarizes our recommendations to promote and build scientific situational awareness.

**Table 2.** Recommendations for Enhancing Scientific Situational Awareness

Recommendation	Example	Considerations
Favor consistency over completeness	Periodic searches in predetermined set of databases or other information sources	Use of standard search terms or keywords and consultation with library science specialists helpful
Value primary sources	Original research publications and/or corresponding original datasets	Prompt reanalysis of data may be warranted if original analysis suspect on scientific or ethical grounds
Prioritize accuracy not primacy	Seek publications that put findings into public health context and provide actionable recommendations	Some journals will not publish claims of primacy
Search beyond original research	Relevant policy statements, legislation, syntheses of expert opinion (eg, from advisory committees)	May require searches for unpublished data or other “grey” literature; library science assistance recommended
Consider curated sources in context	Journals, also current literature awareness services and other information sources where editorial discretion is exercised	Editors bring experience and perspective but are subject to biases; curation increasingly involves technologies such as machine learning, which are not bias-free
Organize in a purpose-built way	Prioritize news databases for communications purposes or legal databases to monitor relevant judicial rulings	Access to specialized databases may require subscriptions or fees
Work across disciplines	Collaboration among various scientific disciplines, health communicators, and information specialists to build scientific situational awareness tools	Different interdisciplinary and multidisciplinary skillsets may be needed across scientific situational awareness functions of searching, curating, and sharing

## Discussion

Awareness of changes in the scientific information environment, along with seeking systematic methods of maintaining scientific situational awareness, are ways to combat the “infodemic” that has been accelerated by COVID-19. The infodemic can be understood as not only the proliferation of misinformation and disinformation, but also as a further acceleration of the sheer quantity of science-related information available, the forms in which it is produced, and the types of audiences seeking to use it. As we prepare for future epidemics, natural disasters, and other public health emergencies, scientific situational awareness—as a unit or function—can be incorporated into emergency operations infrastructure.

In proactively communicating scientific information to enhance the scientific situational awareness of others, the increasing amount of information and concerns about misinformation and disinformation must be considered. For articles that will be published in peer-reviewed literature, authors should write abstracts and summary boxes using plain language principles, such as limiting use of jargon and sentence complexity and length. Abstract or box content may be all that is read or reported. Key messages, which may be incorporated into abstracts or summaries, should be tested for clarity with colleagues, coauthors, or communications experts, keeping in mind educated but inexpert readers, who may include journalists, policymakers, and the interested public, as well as expert and inexpert audiences for whom English is not a first language.

Scientific situational awareness is needed beyond just the issues of the moment. Tools such as current literature awareness services (eg, Science Clips) can be used to actively engage key audiences and stakeholders on important non-COVID-19-related scientific topics, through the use of curated content features. Additional forums for promoting scientific situational awareness can range from individual social media posts to entire journal supplements. Proactive scientific situational awareness can maintain and increase the profile of both infectious disease control priorities, such as ending the HIV epidemic, and chronic disease prevention issues, such as the need for better control of hypertension.^25,26^ Scientific situational awareness as an institutional practice can be used to cut through the “merely urgent” and may be an important way to build institutional trustworthiness.^27^

The public health and broader science community must maintain awareness of the need to monitor for retractions and other corrections to the scientific information environment. One aspect of the digital divide is that not all scientific information is available at the click of a mouse; a great deal of content is still protected by pay walls and embargo policies. Somewhat counterbalancing these concerns about information quality and access is the idea that public health emergencies may spur innovation in meeting scientific information needs, such as the recent within-CDC collaboration to produce a biweekly COVID-19 specific current literature awareness service.^28^

## Conclusion

With the next pandemic or other major public health emergency, the scientific information landscape will likely continue to expand. To publications, preprints, social media, and dashboards will be added means of information sharing that have likely not yet been created. Strategies for maintaining scientific situational awareness will need to continue to evolve, but the work of actively searching for, curating, and seeking to shape scientific information in ways useful to society can begin now. One need is for more tools to organize health-related information, particularly big data, effectively. The efforts to utilize biomedical, biobehaviorial, and other public health relevant data in real time need to be sustained and accelerated beyond health emergencies as a critical aspect of building public health preparedness infrastructure conducive to innovation.
